# Grain boundary structural transformation induced by co-segregation of aliovalent dopants

**DOI:** 10.1038/s41467-022-32935-4

**Published:** 2022-09-15

**Authors:** Toshihiro Futazuka, Ryo Ishikawa, Naoya Shibata, Yuichi Ikuhara

**Affiliations:** 1 Institute of Engineering Innovation, University of Tokyo, Bunkyo, Tokyo, 113-8656 Japan; 2 PRESTO, Japan-Science and Technology Agency, Kawaguchi, Saitama, 332-0012 Japan; 3 Nanostructures Research Laboratory, Japan Fine Ceramics Center, Nagoya, Aichi 456-8587 Japan

**Keywords:** Ceramics, Surfaces, interfaces and thin films, Atomistic models, Transmission electron microscopy

## Abstract

Impurity doping is a conventional but one of the most effective ways to control the functional properties of materials. In insulating materials, the dopant solubility limit is considerably low in general, and the dopants often segregate to grain boundaries (GBs) in polycrystals, which significantly alter their entire properties. However, detailed mechanisms on how dopant atoms form structures at GBs and change their properties remain a matter of conjecture. Here, we show GB structural transformation in *α*-Al_2_O_3_ induced by co-segregation of Ca and Si aliovalent dopants using atomic-resolution scanning transmission electron microscopy combined with density functional theory calculations. To accommodate large-sized Ca ions at the GB core, the pristine GB atomic structure is transformed into a new GB structure with larger free volumes. Moreover, the Si and Ca dopants form a chemically ordered structure, and the charge compensation is achieved within the narrow GB core region rather than forming broader space charge layers. Our findings give an insight into GB engineering by utilizing aliovalent co-segregation.

## Abstract (web summary)

The effect of aliovalent doping on grain boundary is not yet fully understood at the atomic level. Here, the authors report grain boundary structural transformation in α-Al_2_O_3_ is induced by co-segregation of multiple dopants using atomic-resolution electron microscopy and theoretical calculations.

## Introduction

In polycrystalline materials, impurity doping at grain boundaries (GBs) is a major strategy to control physical and chemical properties^1–5^. The dopants with lower solid solubility in bulk are generally segregated to the GBs because GBs usually have a larger volume than the bulk and are able to absorb dopants^6^. When a dopant is isovalent with the bulk constituent atom, the dopants may be segregated to the GBs in a simple substitutional form^4,7,8^. However, for the doping of aliovalent dopants, the charged defects should be formed, which requires other charge-compensating defects such as vacancy, interstitial, or other aliovalent dopants to maintain charge neutrality in the system^9–12^. It is therefore intriguing to elucidate how aliovalent dopants and the counterbalance charged defects are spatially distributed at the GBs, that is, dopants and/or counterbalance charged defects are (i) broadly distributed around the GBs, or (ii) segregated to the GB cores in atomic scale. In the former case, a space-charge layer would be formed to maintain the charge neutrality between the GBs and bulk^13–16^. In the latter case, many charged defects should be accumulated at the GBs, which may cause a large structural transformation of the GB from the pristine case^11^. In this scenario, it is unnecessary to form broad space-charge layers near the GBs, because the charge neutrality can be locally achieved at the GB cores.

Here, we show that the GB structure transformation induced by Ca and Si co-segregation at a Σ13($10\bar{1}4$)/[$11\bar{2}0$] GB in *α*-Al_2_O_3_, by using atomic-resolution scanning transmission electron microscopy (STEM) imaging and spectroscopy, combined with systematic density functional theory (DFT) calculations. Ca and Si dopants in *α*-Al_2_O_3_ are well known to have an impact on the grain growth behavior or the mechanical strength^17^. We then experimentally elucidated that Ca and Si dopants are co-segregated and localized at the Σ13 *α*-Al_2_O_3_ GB core region, and the pristine structure is largely transformed into a new GB structure. To understand the atomistic origin of the GB structure transformation induced by Ca and Si co-segregation, we thoroughly evaluated the GB energy, the segregation energy, and the defect formation energy of Ca and Si dopants at the GB structure by DFT calculations. The calculation results were compared with the atomic-resolution STEM images.

## Results and discussion

### Atomic-resolution STEM imaging of Ca/Si-doped Σ13 *α*-Al_2_O_3_ GB

**Fig. 1:**
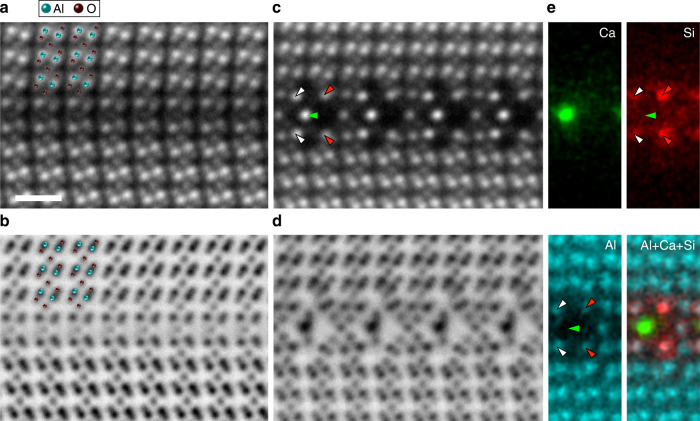
Atomic-resolution STEM images of Σ13($10\bar{1}4$)/[$11\bar{2}0$] GB in *α*-Al_2_O_3_ viewed along the [$11\bar{2}0$] axis. ADF- and ABF-STEM images of **a**, **b** pristine GB and **c**, **d** Ca/Si co-doped GB, respectively. **e** Atomic-resolution STEM-EDS elemental maps of Ca-*K* (green), Si-*K* (red), Al-*K* (cyan) edges, and their overlay, respectively. The green, red, and white arrowheads correspond to the positions of Ca, Si, and weakly observed Si, respectively. The scale bar in a is 5 Å, which applies to all the images.

Figure 1a, b shows simultaneously recorded annular dark-field (ADF) and annular bright-field (ABF) STEM images obtained from the pristine Σ13 *α*-Al_2_O_3_ GB viewed along the $[11\bar{2}0]$ axis, respectively (see Supplementary Fig. 1 for the large field of view)^18,19^. Sequential image averaging was used to improve the signal-to-noise ratio^20^. Owing to the Z-contrast nature (Z is the atomic number) of the ADF image^18^, the heavier Al atomic columns are visualized as brighter dotted contrasts than O atomic columns. In the ABF images, both Al and O atomic columns are well visualized as dark dotted contrasts^19^, and we can determine all the locations of anions and cations at the GB core. The observed atomic structure of the pristine Σ13 GB is consistent with the previously reported G(O) structure^21^, in which the structural units are periodically arranged along the GB as in other high-angle GBs^4,22^. Figure 1c, d shows the ADF- and ABF-STEM images obtained from Ca/Si co-doped Σ13 *α*-Al_2_O_3_ GB viewed along the $[11\bar{2}0]$ axis, respectively. The GB structure is significantly transformed from the G(O) structure. At the center of the GB core (green arrowhead), the bright atomic columns are observed in the ADF image, which could be Ca dopants. To explicitly confirm the distributions of Ca and Si dopants at the Σ13 GB, we performed atomic-resolution STEM-EDS elemental mapping as shown in Fig. 1e. The maps are made by integrating the x-ray counts from Ca-*K* (green), Si-*K* (red), Al-*K* (cyan) edges, and their combination, respectively. We then identified that Ca dopants are segregated only at the center of the GB core, and Si dopants are segregated at the 2^nd^ cation layers (red arrowheads) of the GB core in a mirror-symmetric manner. We note that, in addition to the above atomic sites, Si dopants are slightly segregated at the other atomic sites (white arrowheads). Although there is no EDS signal of Al at the Ca-segregated atomic site, a weak EDS signal of Al is observed at the Si-segregated sites. Therefore, the center of the GB core is fully occupied by Ca dopants, while the second cation layers are shared by Si dopants and Al atoms. According to the image analysis of the sequential ABF-STEM images, there are no significant structural changes and no dynamic behavior of dopant atoms (see Supplementary Note 1), suggesting that the effect of beam damage is negligibly small at the GB core. This is further confirmed by systematic ADF image simulations (see Supplementary Note 3). Our experimental results clearly show that the pristine Σ13 GB structure is transformed into a new GB structure by the co-segregation of Ca and Si dopants at the specific atomic sites of the GB core.

### Determination of the framework of the GB

**Fig. 2:**
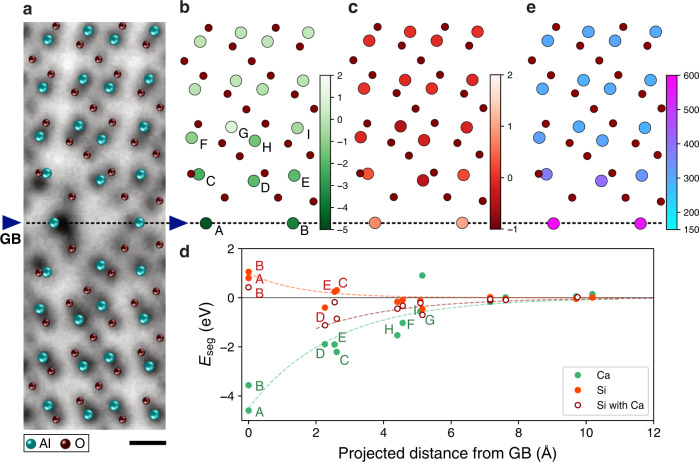
The segregation energies of dopants in pristine Σ13 M(Al) GB. **a** The relaxed atomic structure model overlaid on ABF-STEM image viewed along the $[11\bar{2}0]$ axis, where Al and O atoms are represented by the colors of cyan and dark red, respectively. The arrowheads correspond to the center of the GB core. The dashed line indicates the GB position. The scale bar is 2 Å. The segregation energies of **b** Ca and **c** Si atoms at respective atomic sites in the pristine Σ13 M(Al) GB structure, where the color indicates the segregation energy in eV. **d** The segregation energies of Ca (green), Si (red), and Si with a Ca at A site (white with a dark red edge) as a function of projected distance from the GB plane of ($10\bar{1}4$). The labels of respective atomic sites (A – I) is given in **b**. **e** The Voronoi volumes (Å^3^) at respective cation sites in the structure model of Σ13 M(Al) GB.

To determine the complex atomic structure of the Ca/Si co-segregated Σ13 GB, we firstly explored the framework of the GB atomic structure consisting only of Al and O atoms using the traditional rigid-body translation approach (γ-surface method). However, we could not reproduce the observed GB structure with this method. Therefore, we build up initial structure models by referring to the experimental ADF- and ABF-STEM images. Except for the GB core region, the structure is close to the bulk *α*-Al_2_O_3_, and we consider 256 candidates for the GB structure (see Supplementary Fig. 2). Figure 2a shows the most stable GB structure model of the 256 candidates. The structure model is overlaid on the ABF-STEM image. The GB energy is determined to be 4.17 J m^−2^, which is significantly higher than that of the G(O) structure (2.40 J m^−2^)^21^. Unlike G(O) structure, the present GB plane is mirror-symmetric (in projection), and the GB termination layer is Al atomic column. and hence we hereafter refer to this GB structure as M(Al). Although Ca and Si dopants are not introduced in this structure model, the atomic positions matched those in the ABF-STEM image, except at the GB core. The calculated GB energy of the M(Al) structure is much higher than that of the G(O) structure, suggesting that M(Al) structure cannot be stably formed without Ca/Si co-segregation.

### Segregation energies of Ca and Si at M(Al) GB

Prior to exploring the most energetically stable Ca/Si co-segregated M(Al) GB structure, we systematically calculated segregation energies of individual Ca and Si dopants in the M(Al) GB structure. Figure 2b, c shows the segregation energies of Ca and Si dopants for each atomic site in the GB, where we evaluated the segregation energies of ${{{{{{\rm{Ca}}}}}}}_{{{{{{\rm{Al}}}}}}}^{1-}$ and ${{{{{{\rm{Si}}}}}}}_{{{{{{\rm{Al}}}}}}}^{1+}$ at 17 Al sites (see Fig. 2b for the labels of atomic sites). It is noteworthy that the valence states of Ca and Si for Al substitutional defect in the bulk should be 2+ and 4+ (ref. 23), and the valence state of Ca in the GB core was confirmed to be 2+ by electron energy-loss spectroscopy (see Supplementary Note 2). Figure 2d shows the segregation energy of Ca and Si as a function of the projected distance from the GB plane. The Ca segregation energy decreases from the bulk to the GB and be minimized at the A site (–4.63 eV), which is well consistent with the STEM-EDS results of Fig. 1e. The Ca segregation energy at the A site is very low and hence Ca segregation at the GB core should strongly contribute to the structural stability of M(Al) GB structure. Although the Si segregation energies in the second (D site, –0.38 eV) and third (G site, –0.46 eV) cation layers are slightly lower than those of the others, the site dependence of the Si segregation energy is much smaller than that of Ca. Moreover, the segregation energy of Si increases from the bulk to the GB, suggesting that Si should not be solely segregated to the M(Al) GB structure. Therefore, we further calculated Si segregation energy with a Ca dopant fixed at the A site, as shown in Fig. 2d. Compared with the Si sole segregation, the Si segregation energies with a Ca at the A site are overall reduced and minimized in the second cation layers (C site: –0.85 eV, D site: ‒1.11 eV), which is consistent with the STEM-EDS results. These results suggest that Si dopants can also contribute to stabilizing the M(Al) GB structure by segregating to the neighbors of Ca dopant, and the charge compensation will be achieved within the GB core, rather than forming space-charge layers.

Since the ionic radius of Ca (100 pm) is much larger than that of Al (53.5 pm)^24^, the larger free volume at the GB core should be required for accommodating Ca dopants. Figure 2e shows the Voronoi volumes for respective atomic sites. The Voronoi volumes at A and B sites are two times larger than that in bulk, and hence the Ca segregation energy is significantly reduced at the A and B sites. The segregation energies of Si with a Ca at the A site are evidently reduced at C and D sites, whose Voronoi volumes are the second largest among atomic sites. However, the ionic radius of Si (40 pm) is smaller than that of Al, and hence the large Voronoi volume is not required for Si segregation. The atomic environments of C and D sites are tetrahedral coordination, which is different from the octahedral coordination of Al in bulk. Si dopants prefer to form covalent bonds with the neighboring O atoms (well known as SiO_2_ tetrahedron), and therefore the Si segregation energies are reduced by the tetrahedral coordination at C and D sites^23^.

### Determination of Ca/Si co-segregated Σ13 GB structure

**Fig. 3:**
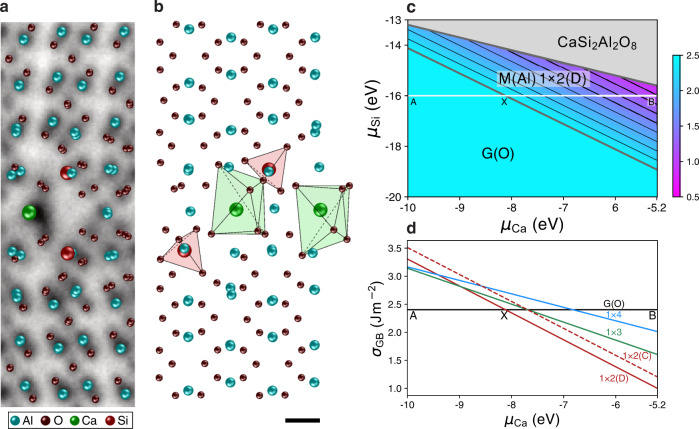
The relaxed atomic structure and the GB energy of Ca/Si co-doped Σ13 M(Al) GB. The structure model is viewed along the **a** $[11\bar{2}0]$ axis and **b** $[\bar{2}021]$ axis, where the structure model in **a** is overlaid on the ABF-STEM image. Al, O, Ca, and Si atoms are represented by the colors cyan and dark red, green, and light red, respectively. The structural tetrahedron (pale red) and octahedron (pale green) for Si and Ca are given in **b**. The scale bar in **b** is 2 Å, which applies to **a**. **c** The GB energy *(*J m^−^^2^) of Σ13 *α*-Al_2_O_3_ GB in an oxygen-rich atmosphere as functions of chemical potentials of Ca and Si. At maximum chemical potentials of Ca and Si, CaAl_4_O_7_ and Al_2_SiO_5_ are formed, respectively. **d** The cross-sectional view of the GB energy along the white line of A-X-B in **c**.

Finally, we determine the most energetically stable Ca/Si co-segregated M(Al) GB structure by referring to the STEM experiments and the theoretical segregation energies of Ca and Si in the M(Al) GB structure. The most preferable location of ${{{{{{\rm{Ca}}}}}}}_{{{{{{\rm{Al}}}}}}}^{1-}$ defect can be fixed at A site, which is fully occupied by Ca dopants. For Si dopants, we consider two atomic sites (C and D sites). To maintain the charge neutrality in the system, the number of ${{{{{{\rm{Ca}}}}}}}_{{{{{{\rm{Al}}}}}}}^{1-}$ defects should be equal to that of ${{{{{{\rm{Si}}}}}}}_{{{{{{\rm{Al}}}}}}}^{1+}$. Although the Si dopants are segregated to the second layer of the GB in a mirror-symmetric manner (in projection), we experimentally confirmed that the corresponding atomic sites (C or D sites) are shared by Si dopants and Al atoms. Therefore, we construct the superstructure along the depth direction, i.e., 1 × 2, 1 × 3, and 1 × 4 supercells. When the atomic sites of Ca and Si are fixed at A and D sites, respectively, the numbers of possible candidates are 2, 6, and 22 for 1 × 2, 1 × 3, and 1 × 4 superstructures, respectively (See Supplementary Figs. 3,4). It is noteworthy that we also consider the partial occupation of Si at the C site (shared with Al atoms) for 1 × 3 and 1 × 4 superstructures. On the basis of these systematic calculations, the most stable Ca/Si co-segregated M(Al) GB structure is determined to be a 1 × 2 superstructure with the Si dopants located at the D site (we denote as 1 × 2(D)), as shown in Fig. 3a. Compared with the pristine M(Al) GB structure of Fig. 2a, all the atomic positions of 1 × 2(D) superstructure are excellently matched with those in the ABF-STEM image, especially at the GB core region. As shown in Fig. 3b viewed along the [$\bar{2}021$] axis (orthogonal direction of Fig. 3a), the Si dopant distribution in three dimensions is not mirror-symmetric but alternatively segregated along the projection across the GB. As noticed in Fig. 2e, we can confirm that the Ca dopant is stable at the largest Voronoi space and Si dopants are stable at the SiO_2_ tetrahedral coordination sites. Figure 3c shows the GB energy diagram of the most stable structures as functions of chemical potentials of Ca and Si. An impurity-doped GB is non-stoichiometric, and thus the GB energy varies with the chemical potentials of µ_Ca_ and µ_Si_ in a whole system. At the low µ_Ca_ and µ_Si_ region (low concentrations of Ca and Si in bulk), the most stable GB structure is the pristine G(O), in which the Ca/Si dopants would not be segregated to the GB, but rather be dissolved in bulk. As the dopant chemical potentials increase, Ca and Si dopants should be segregated to the GB, and the framework of the GB structure is transformed from G(O) to M(Al). For the further increment of the dopant chemical potentials, the CaSi_2_Al_2_O_8_ phase would be precipitated at the GB. Figure 3d shows the cross-sectional view of the GB energy as a function of µ_Ca_ (µ_Si_ = –16 eV) along the white A–X–B line. At the point X, the most stable GB structure is transformed from G(O) to the Ca/Si co-segregated 1 × 2(D) superstructure, and the GB energy becomes less than 2.40 J m^−2^. The 1 × 3 and 1 × 4 superstructures are less stable than either G(O) or 1 × 2(D) GB structures in all the chemical potential range. It is noteworthy, that in our present annealing condition, the GB structure may not reach the complete thermal equilibrium, and therefore the second most stable 1 × 2(C) superstructure may be weakly observed in the experiment.

**Fig. 4:**
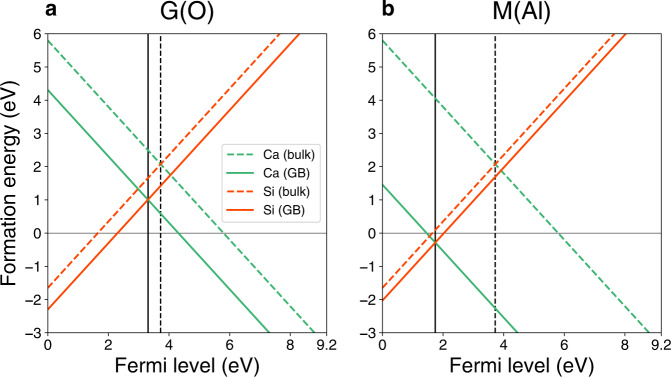
The defect formation energies of Ca and Si in bulk (dashed lines) and at the Σ13 α-Al_2_O_3_ GB. Defect formation energies at **a** G(O) and **b** M(Al) GB structures (solid lines) as a function of Fermi level, respectively. The Ca and Si dopants are located at A and D sites in the M(Al) GB structure, respectively. The black vertical lines correspond to the Fermi levels at respective thermal equilibrium.

To understand the origin of the GB structure transformation induced by the Ca/Si co-segregation at the GB, we further calculated the defect formation energies of ${{{{{{\rm{Ca}}}}}}}_{{{{{{\rm{Al}}}}}}}^{1-}$ and ${{{{{{\rm{Si}}}}}}}_{{{{{{\rm{Al}}}}}}}^{1+}$ as a function of Fermi level in both the M(Al) and G(O) GB structures. Figure 4a, b shows the defect formation energies of ${{{{{{\rm{Ca}}}}}}}_{{{{{{\rm{Al}}}}}}}^{1-}$ (A site) and ${{{{{{\rm{Si}}}}}}}_{{{{{{\rm{Al}}}}}}}^{1+}$ (D site) in the M(Al) or the most stable site in the G(O) GB structures, respectively. Dashed and solid lines indicate the defect formation energies as a function of the Fermi level in bulk and at the GB, respectively. We assumed that charge neutrality is achieved by the combination of ${{{{{{\rm{Ca}}}}}}}_{{{{{{\rm{Al}}}}}}}^{1-}$ and ${{{{{{\rm{Si}}}}}}}_{{{{{{\rm{Al}}}}}}}^{1+}$. In thermal equilibrium, the Fermi levels correspond to the intersection points between solid and dashed lines, respectively, as indicated by black vertical solid and dashed lines in Fig. 4, and hence the defect formation energies of ${{{{{{\rm{Ca}}}}}}}_{{{{{{\rm{Al}}}}}}}^{1-}$ and ${{{{{{\rm{Si}}}}}}}_{{{{{{\rm{Al}}}}}}}^{1+}$ must be equal at the Fermi level. Here, we consider the defect formation energies of Ca and Si at the Fermi level. In the bulk of *α*-Al_2_O_3_, the defect formation energy of Ca/Si is 2.0 eV. On the other hand, the defect formation energies of Ca/Si at the G(O) and M(Al) GBs are reduced to 1.0 and –0.43 eV, respectively. At the G(O) GB, the defect formation energy of Ca/Si is still relatively large, and hence the Ca/Si concentration at the G(O) should be very low. However, at the M(Al) GB, the defect formation energy of Ca/Si becomes negative, and hence Ca/Si co-segregation at the M(Al) GB would be spontaneously promoted. Although the difference in the Si segregation energy between the M(Al) GB and the bulk is considerably small, the Fermi level is significantly reduced from 3.3 to 1.6 eV because of the large gain of the Ca segregation energy at the M(Al) GB. Therefore, the defect formation energy of ${{{{{{\rm{Si}}}}}}}_{{{{{{\rm{Al}}}}}}}^{1+}$ also becomes negative.

In summary, we have investigated the structural transformation of Σ13 *α*-Al_2_O_3_ GB induced by the co-segregation of Ca and Si aliovalent dopants with the aid of atomic-resolution STEM imaging and spectroscopy combined with DFT calculations. To accommodate Ca dopants with larger ionic radius and Si with strong covalency, the pristine G(O) GB structure is transformed into a new type of M(Al) GB structure, where the structural unit has a large free volume and tetrahedral environments. Both the aliovalent ${{{{{{\rm{Ca}}}}}}}_{{{{{{\rm{Al}}}}}}}^{1-}$ and ${{{{{{\rm{Si}}}}}}}_{{{{{{\rm{Al}}}}}}}^{1+}$ dopants are segregated to the GB core region, and hence the charge neutrality is achieved at the GB core region, rather than forming space-charge layers around the GB. This is also confirmed by the further macroscopic electrostatic calculations, where we found the formation of charged GB and the space-charge layer is unfavorable (see Supplementary Note 4). Our findings indicate that aliovalent co-doping could be a new strategy to locally segregate multiple dopants, and it becomes possible to tailor a new functional GB atomic structure.

## Methods

### Sample preparation and electron microscopy

We fabricated pure and Ca/Si co-doped Σ13($10\bar{1}4$)/[$11\bar{2}0$] GBs in *α*-Al_2_O_3_ by thermal diffusion bonding of two single crystals at 1773 K for 10 h in the air. Ca and Si are doped to the GB plane before diffusion bonding in the aqueous forms of Ca(CH_3_COO)_2_ and SiO_2_, where these aqueous concentrations are 0.005 mol L^−1^, and thus, Ca and Si are initially doped with the same concentration. It is thus considered that charge neutrality is naturally preserved by alternate co-segregation of aliovalent dopants as the grain boundary is formed. At this condition, we observed no dopant-related precipitations at the GB. TEM specimen was prepared by mechanical polishing and Ar ion milling. Atomic-resolution STEM imaging and EDS mapping were acquired with a JEM ARM200CF (JEOL Ltd.), operated at 200 kV. To suppress electron beam damage, the typical probe current is set to ~9 pA. Under the present acquisition condition, no significant image contrast changes in ADF/ABF-STEM images were observed (see Supplementary Note 1). The probe forming aperture was 24 mrad in semi-angle, and ADF and ABF collection semi-angles were spanned 12–24 mrad and 64–200 mrad, respectively. EEL spectra were obtained by a Continuum spectrometer (Gatan Inc.) equipped with the ARM200CF microscope, where the energy dispersion was 0.3 eV per channel. These EEL spectra were recorded by a box scanning mode along the GB core to suppress electron beam damage (the beam current was ~10 pA).

### Theoretical calculations

The calculations were performed using the projector augmented wave method implemented in the Vienna ab initio simulation package (VASP^25–27^) code with GGA-PBEsol functional^28^. The GB energy ${\sigma }_{{{{{{\mathrm{GB}}}}}}}$ is calculated using the following equation:

1 $${\sigma }_{{{{{{\mathrm{GB}}}}}}}=\frac{1}{2A}({E}_{{{{{{\mathrm{GB}}}}}}}-{\sum }_{i}{n}_{i}{\mu }_{i})$$

where ${E}_{{{{{{\mathrm{GB}}}}}}}$ is the total energy of the supercell with two GBs. ${n}_{i}$ is the number of the element $i$ in the supercell, and ${\mu }_{i}$ is the chemical potential of the element $i$. The segregation energy of a dopant is defined as the difference between defect formation energy at *j* site of the GB and in the bulk:

2 $${E}_{{{{{{\mathrm{seg}}}}}}}[j]={E}_{f}^{{{{{{\mathrm{GB}}}}}}}[j]-{E}_{f}^{{{{{{\mathrm{bulk}}}}}}}\,\cong \,{E}_{{{{{{\mathrm{tot}}}}}}}^{{{{{{\mathrm{GB}}}}}}}[j]-{E}_{{{{{{\mathrm{tot}}}}}}}^{{{{{{\mathrm{bulk}}}}}}}$$

where we approximate the segregation energy by ignoring the cell size dependence. Both the GB energy and the segregation energy depend on the supercell size.

## Supplementary information

Supplementary Information

## Data Availability

The presented data were available from the corresponding author upon request.
