# Supplementary material for: Grain boundary structural transformation induced by co-segregation of aliovalent dopants
Source: Nat Commun. 2022 Sep 15;13:5299. doi: 10.1038/s41467-022-32935-4 (PMC9477882; doi:10.1038/s41467-022-32935-4)
Supplement: Supplementary file 1 — Supplementary Information [file 41467_2022_32935_MOESM1_ESM.pdf]

Supplementary Information

Grain boundary structural transformation induced by co-segregation of aliovalent dopants

Futazuka et al.

Grain boundary structural transformation induced by co-segregation of aliovalent dopants

Toshihiro Futazuka[1], Ryo Ishikawa[1,2,*], Naoya Shibata[1,3], Yuichi Ikuhara[1,3,*]

[1]Institute of Engineering Innovation, University of Tokyo, Bunkyo, Tokyo, 113-8656, Japan

[2]PRESTO, Japan-Science and Technology Agency, Kawaguchi, Saitama 332-0012, Japan

[3]Nanostructures Research Laboratory, Japan Fine Ceramics Center, Nagoya, Aichi, 456-8587, Japan

*ishikawa@sigma.t.u-tokyo.ac.jp (R.I.), ikuhara@sigma.t.u-tokyo.ac.jp (Y.I.)

Supplementary Note 1. To check the structural stability of the Ca/Si co-doped Σ13 α-Al_2O_3 GB under the electron beam irradiation, we investigate the variations of the image contrast in the sequentially acquired ABF-STEM images. In our experiments, to suppress electron beam damage, we acquired 30 frames of ABF-STEM images with relatively low beam current (~9 pA) and fast scanning of the probe (4 μs/pix). Therefore, the noise level for respective frames is considerably high. We therefore applied a 2D Gaussian filter to enhance the visibility of individual frames. Supplementary Figure 5 shows the selected frames (every two frames) of a series of ABF-STEM images obtained from the GB core region. No significant image contrast changes were observed. This result suggests that the number of lattice defects such as vacancies should be negligibly small in the present GB core region, and Ca and Si could be fully occupied at respective atomic sites.

Supplementary Note 2. In oxides, the oxidation states of Ca and Si are usually 2+ and 4+, respectively, and the other oxidation states may not be formed. This is also valid for Al substitutional defects of Ca and Si in α-Al_2O_3 [1]. In order to experimentally confirm the oxidation states of Ca and Si at the GB, we performed STEM-EELS, and obtained the EEL spectra from the bulk and GB as shown in Supplementary Figure 6a. Two distinct edges were observed, *i.e.*, Al-$L_{2,3}$ edges at 80 eV and Ca-$L_{2,3}$ edges at 350 eV, where the weak signal of Ca-$L_{2,3}$ edges can be found only at the GB. Although Si dopants should also be localized at the GB

core, we could not find Si L-edges at the GB core. This is because the strong Al-$L_{2,3}$ and the related minor edges are completely overlapped with the Si-$L_{2,3}$ edge. Therefore, it is difficult to experimentally confirm the electronic structure of Si at the GB core. It is noteworthy that all the peaks of Al at the GB core become broader than that in the bulk, which is the most evident in the onset of Al-L_3 edge, as indicated by the arrows in Suppl. Fig. 5b. The downward energy shift is 1.2 eV, corresponding to the lower coordination number at the GB core [2]. Supplementary Figure 6c shows the Ca-$L_{2,3}$ edge at the GB core, and the profile is similar to that of CaO [3], suggesting that the oxidation state of Ca should be 2+ ($\mathrm{Ca}_{\mathrm{Al}}^{1-}$) at the GB core.

Supplementary Note 3. To evaluate the Ca occupation at the site A (Fig. 2), we performed multi-slice image simulations using the frozen phonon model with a 200 kV electron probe, illumination semi-angle of 24 mrad, and an ADF detector spanning 64 to 150 mrad [4]. The frozen phonon calculation assumed an Einstein model and used 10 phonon configurations. On the basis of the log-ratio method in EELS, the specimen thickness was estimated to be 15 ± 3 nm, and we assumed the specimen thickness as 14.2 nm in our image simulation, which contains 30 atoms in the Ca atomic column. There are too many configurations along the projection when considering the multiple Al sites at the A site. Therefore, we used the fractional atomic potential for Ca rather than considering the configuration of $\mathrm{Ca}_{\mathrm{Al}}^{1-}$. Suppl. Fig. 7a shows the experimental and simulated ADF-STEM images with the Ca occupation between 50% and 100%, and the Z-contrast intensity at the Ca atomic column increases as a function of Ca occupation. Suppl. Fig. 7b shows the experimental and simulated Z-contrast intensity profiles along the A-B direction, where the intensities are normalized to the Al atomic column as indicated by the red arrowhead. The error bars in the experimental profile corresponds to standard deviation, which were calculated from the 5 different line profiles. Comparing the experiment with simulations, the Ca occupation may be estimated to be 90 ± 10%, suggesting that the Ca atomic column could be almost fully occupied. We note that the present comparison

was performed in the relative intensity rather than the absolute-intensity scale, and therefore the reliability of this quantification may be limited.

Supplementary Note 4. To investigate the possible formation of the charged GB and the space-charge layer, we performed macroscopic electrostatic calculations using the standard Gouy-Chapman model. As an example, we considered the positively charged M(Al) 1×1 GB with one $\mathrm{Ca}_{\mathrm{Al}}^{1-}$ and two $\mathrm{Si}_{\mathrm{Al}}^{1+}$ in the GB structure unit. In this case, we assumed that $\mathrm{Ca}_{\mathrm{Al}}^{1-}$ would distribute around the positively charged GB core (originate from the extra $\mathrm{Si}_{\mathrm{Al}}^{1+}$) to form the space-charge layer. We evaluated the electrostatic potential and the $\mathrm{Ca}_{\mathrm{Al}}^{1-}$ concentration profiles in the space-charge layer as follows. When the positively charged GB is formed, the electrostatic potential around the GB should be higher than that in the bulk, and we denote this extra electrostatic potential as $\phi(x)$ (x: distance from the GB core). Using the Boltzmann distribution with the defect formation energy of $\mathrm{Ca}_{\mathrm{Al}}^{1-}$ in the bulk, the $\mathrm{Ca}_{\mathrm{Al}}^{1-}$ concentration profile $c(x, T)$ can be written as

$$c(x,T) = c_b\,(T)exp\left(-\frac{q\phi(x)}{kT}\right), \qquad (1)$$

where $c_b(T)$ is the solubility limit of $\mathrm{Ca}_{\mathrm{Al}}^{1-}$ in the bulk at an absolute temperature T, and k is the Boltzmann constant. The electrostatic potential $\phi(x)$ in the space-charge layer can be evaluated by solving the Poisson's equation with the boundary condition:

$$\frac{d^2\phi(x)}{dx^2} = -\frac{1}{\epsilon}qc(x,T), \qquad (2)$$

$$\left(\frac{d\phi(x)}{dx}\right)_{x=\infty} = 0, \qquad (3)$$

where ϵ is the permittivity of α-Al_2O_3. These equations of (1) – (3) can be transformed into the following differential equation:

$$\frac{d\phi(x)}{dx} = -\sqrt{\frac{2kT}{\epsilon}\left(c(x,T) - c_b(T)\right)}. \qquad (4)$$

The electrostatic potential around the GB $\phi(x)$ is determined self-consistently to satisfy the

following charge-neutrality between the GB core and the space-charge layer:

$$q_{GB} + 2\int_0^{\infty}(c(x,T) - c_b(T))\,dx = 0, \quad (5)$$

where q_{GB} is the charge density at the GB. By numerically solving the differential equation (4), we evaluated the electrostatic potential $\phi(x)$ and the $\mathrm{Ca}_{\mathrm{Al}}^{1-}$ concentration $c(x,T)$. Suppl. Figs. 8a and 8b show the electrostatic potential and the $\mathrm{Ca}_{\mathrm{Al}}^{1-}$ concentration at the sintering temperature of 1773 K, respectively. Although the electrostatic potential spans several μm from the GB, the $\mathrm{Ca}_{\mathrm{Al}}^{1-}$ is localized within only a few Å from the GB core, *i.e.*, 98.4 % of the $\mathrm{Ca}_{\mathrm{Al}}^{1-}$ is localized within 5 Å from GB core. This result strongly suggests that the formation of charged GB and the space-charge layer is unfavorable, but the charge compensation would be achieved locally within the GB core.

Supplementary References

1. Futazuka, T. *et al.* First-principles calculations of group IIA and group IV impurities in α-Al_2O_3. *Phys. Rev. Mater.* **4**, 073602 (2020).
2. Wei, J. *et al.* Direct Measurement of Electronic Band Structures at Oxide Grain Boundaries. *Nano. Lett.* **4**, 2530-2536 (2020).
3. Gu, H. *et al.* Indirect EELS imaging reaching atomic scale -CaO planar faults in $CaTiO_3$. *Ultramicroscopy* **78**, 221-231 (1999).
4. Kirkland, E. J. *Advanced Computing in Electron Microscopy. Springer* (2020). doi: 10.1007/978-3-030-33260-0.

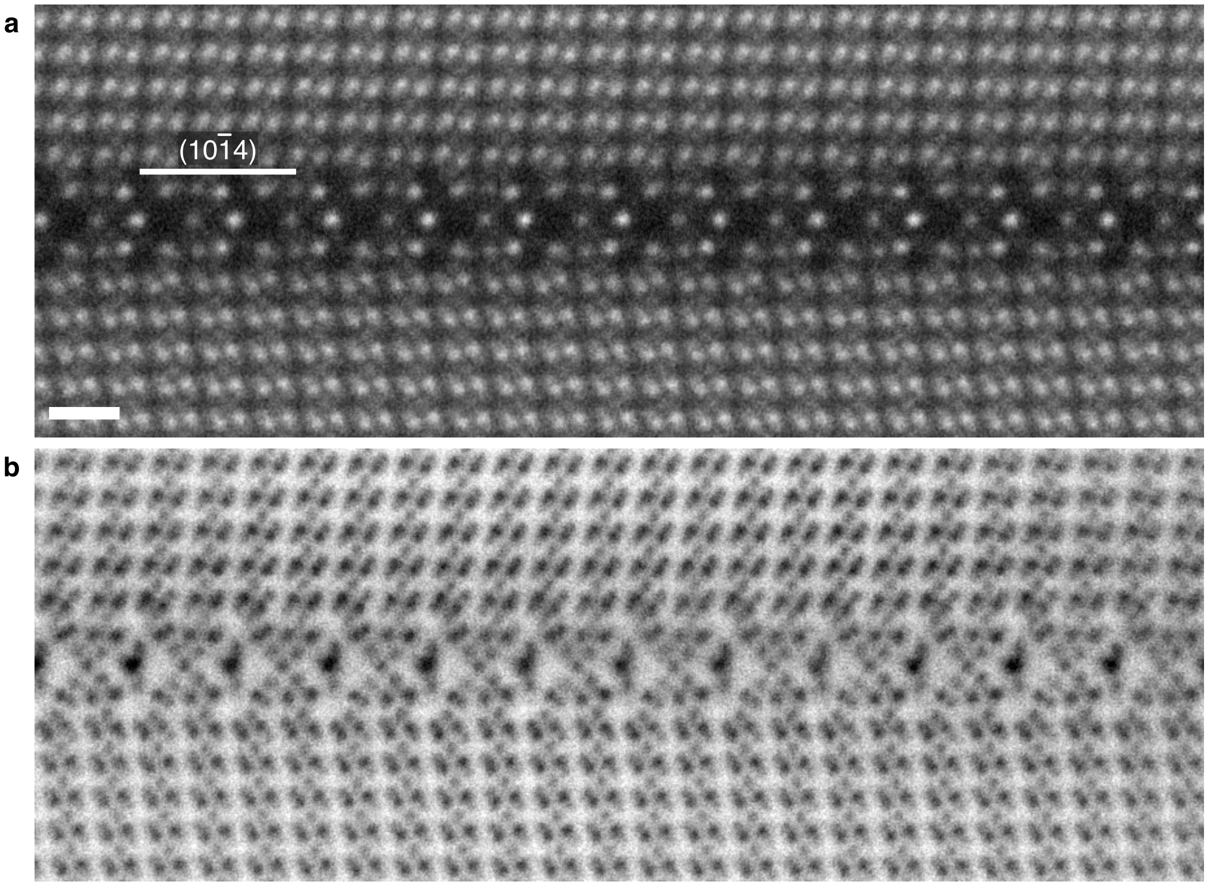

Supplementary Fig. 1 Large field-of-view STEM images of Ca/Si doped Σ13 grain boundary (GB). **a** ADF and **b** ABF-STEM image of GB viewed along the [11$\bar{2}$0] axis, respectively. The scalebar in **a** is 5 Å, which is applied to all images in Supplementary Fig. 1.

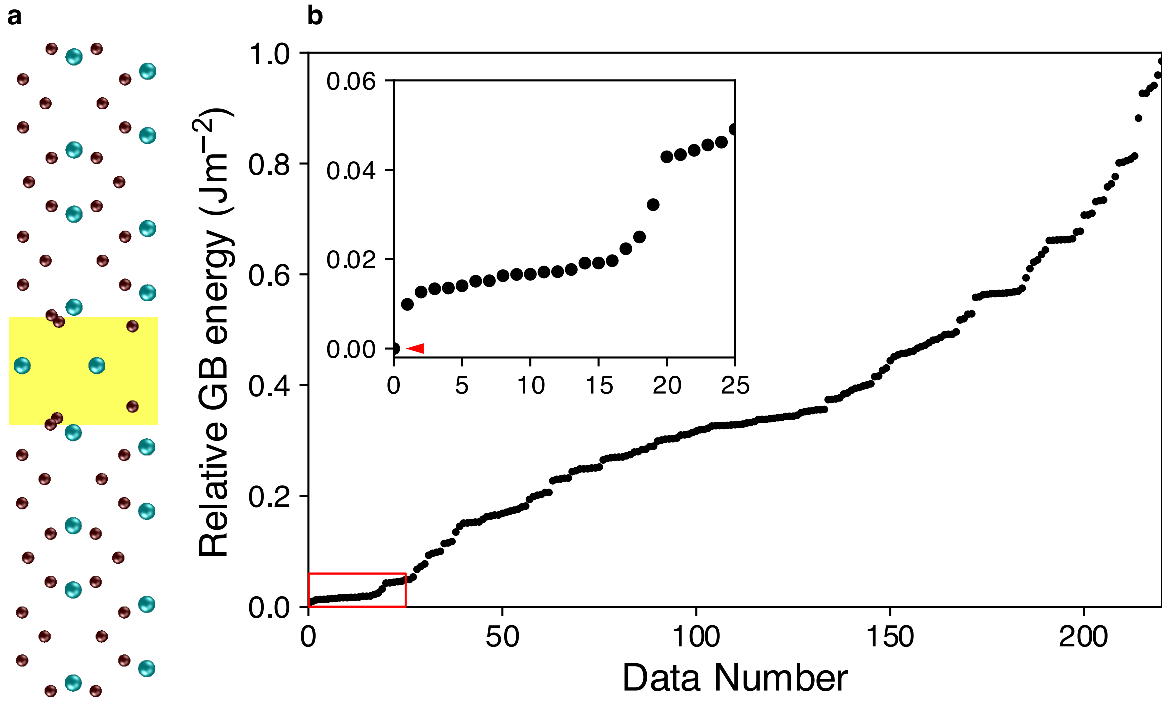

Supplementary Fig. 2 The grain boundary (GB) energies of pristine M(Al) GBs. a One of initial structure models of M(Al) GB viewed along the [$\bar{2}$021] axis. Six atoms including two Al and four O in the yellow region are significantly deviated from the bulk configurations. **b** The relative GB energies for all the explored GB structure models, where we calculated 256 GB candidates. The zero in relative GB energy corresponds to the GB energy of the most stable M(Al) GB structure. Red rectangle region is magnified and shown as inset.

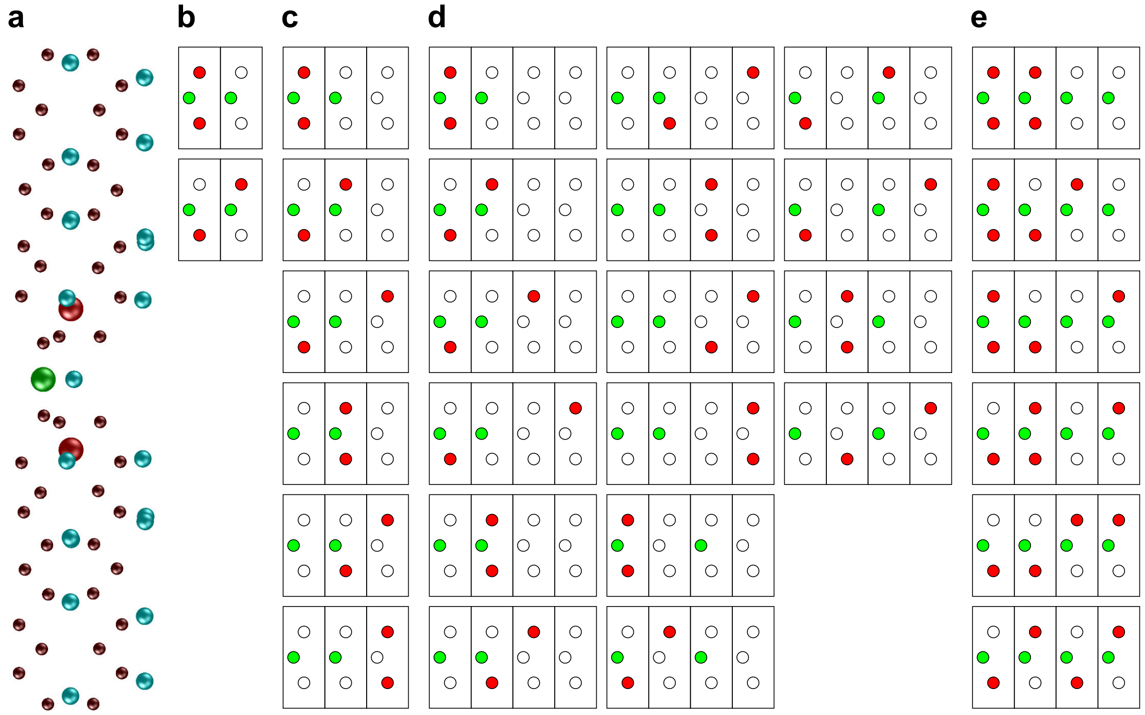

Supplementary Fig. 3 The candidate structures for Ca/Si co-doped M(Al) grain boundary (GB). a The candidate positions for the segregation sites of Ca/Si in 1×1 GB supercell viewed along the $[\bar{2}021]$ axis. The number of possible segregation sites in the GB unit cell are one and two for Ca and Si, respectively. The schematic pictures of all the candidates of Ca and Si configurations along the depth, where only the Ca (green) and Si (red) are shown: **b** 1×2, **c** 1×3, **d** 1×4 supercells with two Ca/Si, and **e** 1×4 supercell with four Ca/Si.

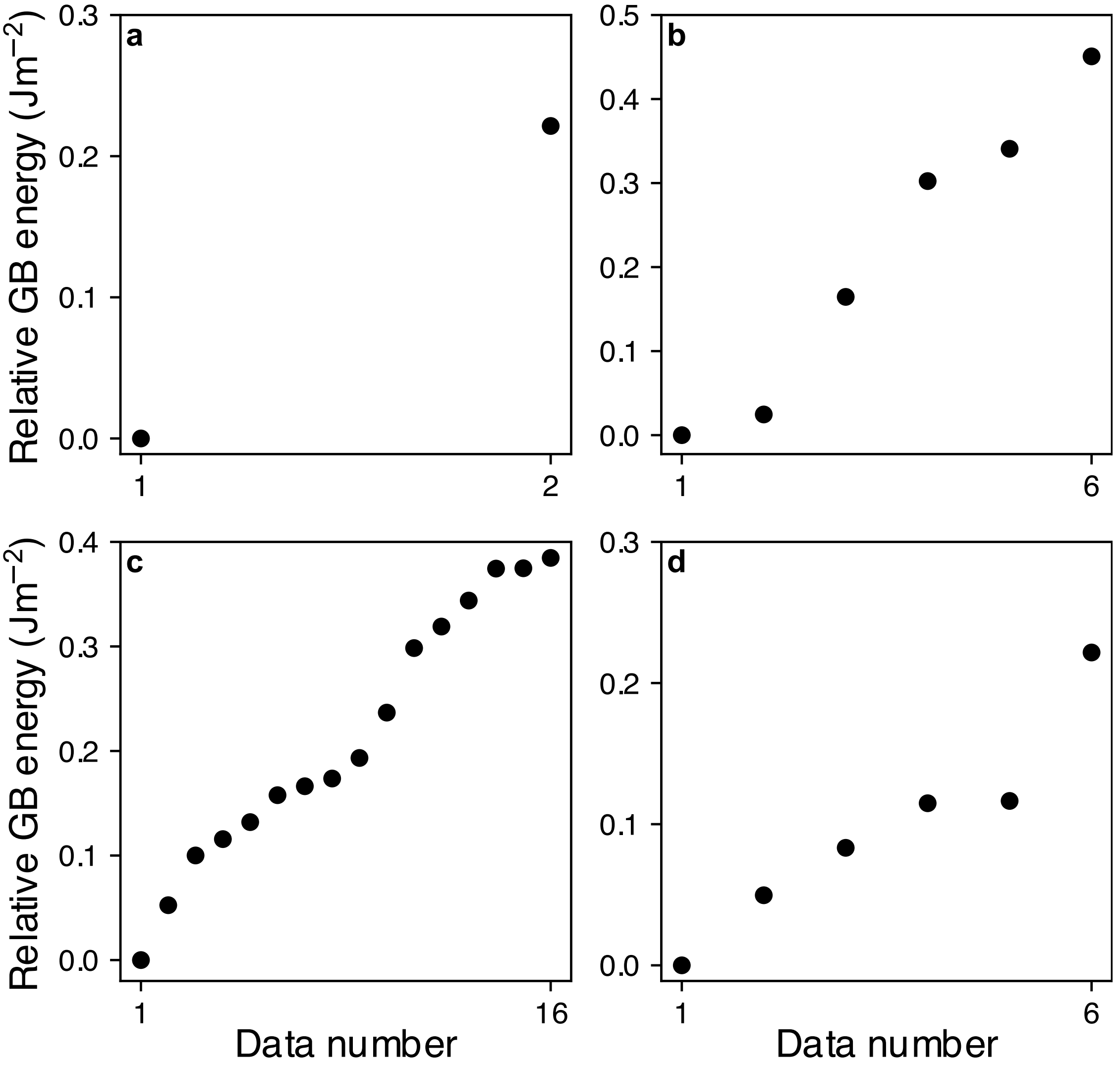

Supplementary Fig. 4 The relative grain boundary (GB) energies for all candidates of superstructures. GB energies for **a** 1×2(D), **b** 1×3(D), **c** 1×4(D) superstructures with two Ca/Si, and **d** 1×4 superstructure with four Ca/Si, respectively.

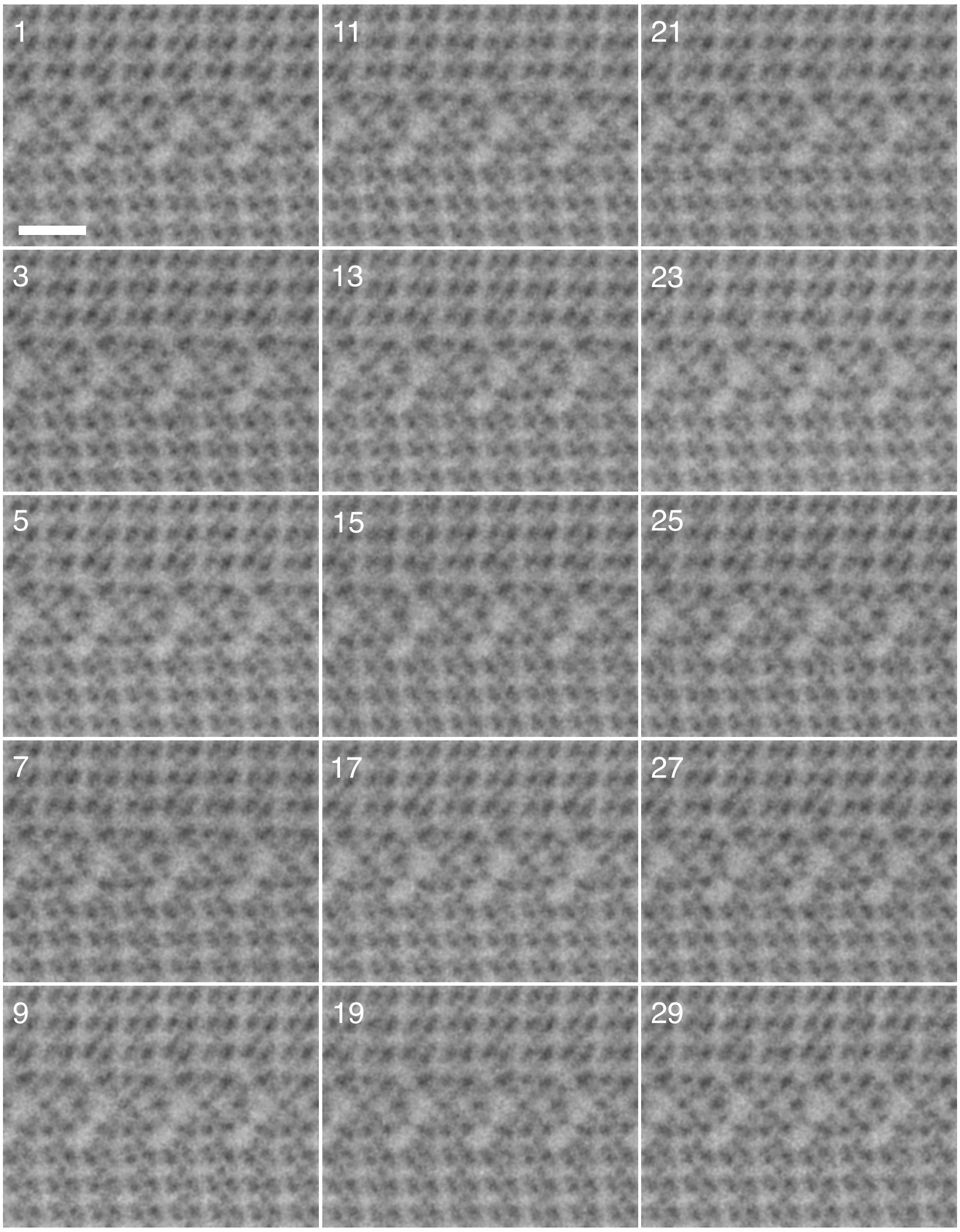

Supplementary Fig. 5. A sequence of ABF-STEM images. Selected frames from a sequence of 30 ABF-STEM images, and the frame numbers are given in each panel. The scale bar in the left-top panel is 5 Å, which is applied to all images in Supplementary Fig. 5.

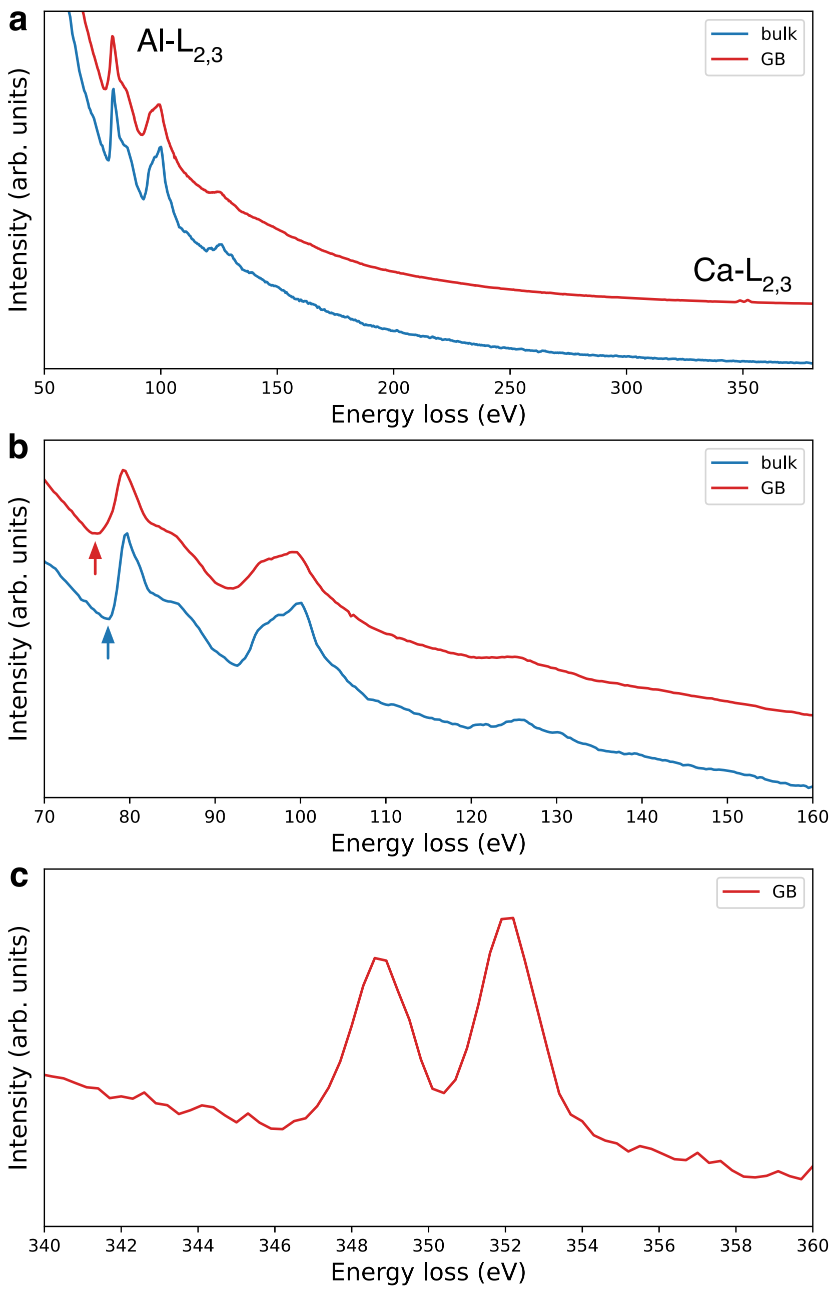

Supplementary Fig. 6. EELS spectra obtained from the bulk and at the grain boundary (GB) core. a EEL spectra obtained from the GB and the bulk. **b** Al-$L_{2,3}$ edges obtained from the GB and the bulk, and **c** Ca-$L_{2,3}$ edges obtained from the GB.

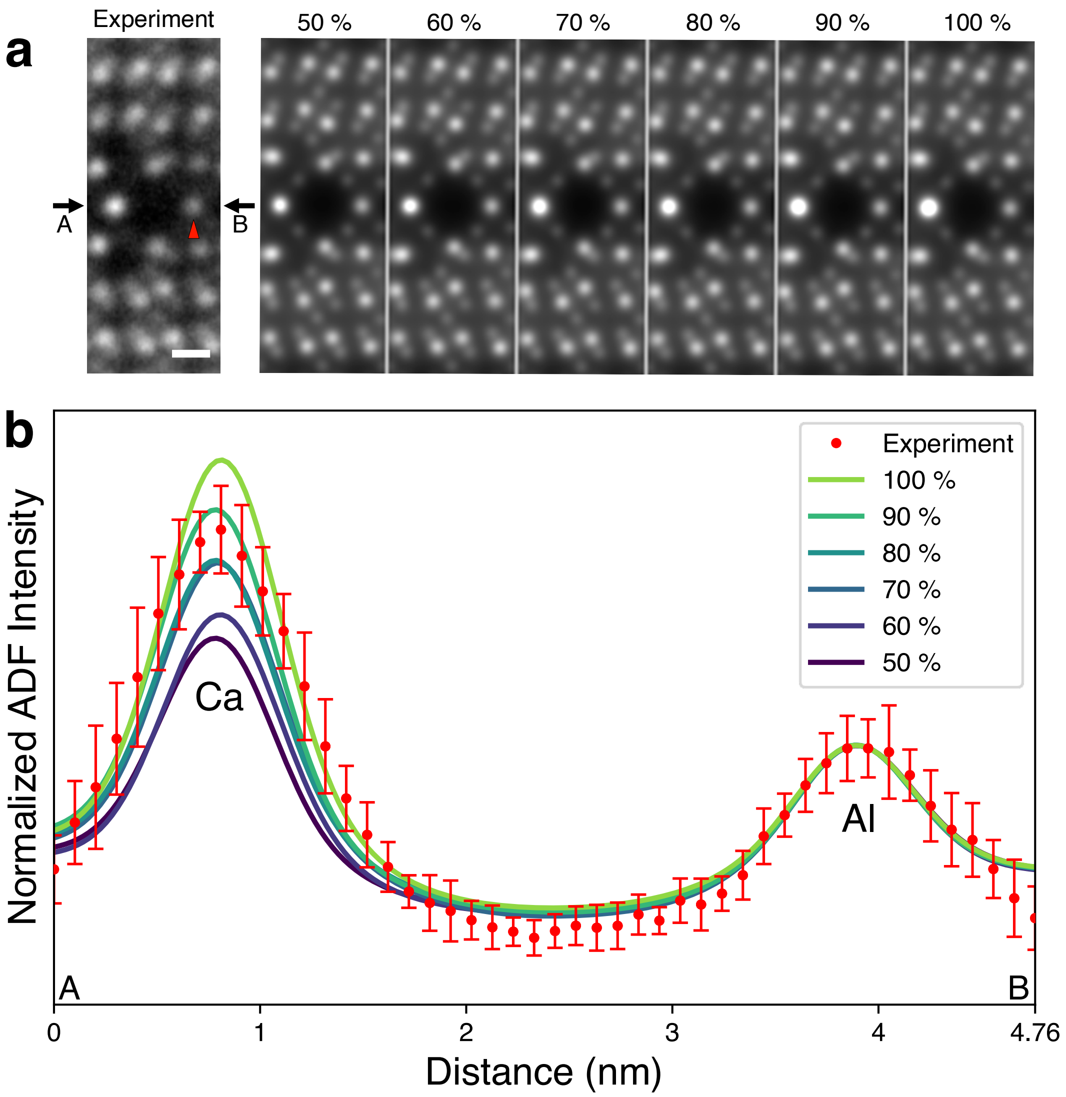

Supplementary Fig. 7 Simulated ADF-STEM images with different Ca occupations. a The experimental and simulated ADF-STEM images. The occupation ratio of Al-substitutional Ca site ranges from 50% to 100%. The scale bar is 2 Å, which is applied to all images in Supplementary Fig. 7. **b** The ADF intensity profiles along the A-B direction in **a**, where the intensities are normalized to the Al atomic column (red arrow in **a**). The error bars correspond to the standard deviations for intensities of eleven structural units along the GB.

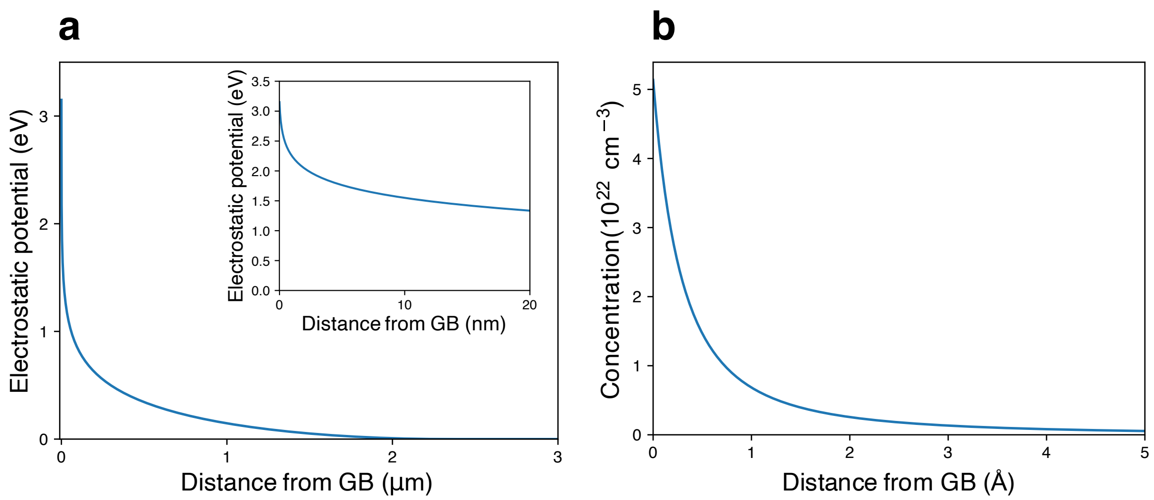

Supplementary Fig. 8 The potential profile for charged M(Al) grain boundary (GB). a The electrostatic potential profile for the charged M(Al) GB (one extra Si dopant). The inset is the magnified profile within 20 nm from the GB. **b** The concentration profile of Ca_{Al}^{1-} as a function of distance from the GB.
